# Wound-healing, anti-inflammatory, and antioxidant activities of β-glucan from red sea-mangroves-associated *Candida tropicalis*

**DOI:** 10.1038/s41598-026-42067-0

**Published:** 2026-04-01

**Authors:** Aya T. ElGazzare, Basma M. Alkersh, Soraya A. Sabry, Hanan A. Ghozlan

**Affiliations:** 1 Department of Botany and Microbiology, Faculty of Science, Alexandria University, Alexandria, Egypt; 2 National Institute of Oceanography and Fisheries (NIOF), Alexandria, Egypt

**Keywords:** Marine yeast, β-glucan, *Candida tropicalis*, Antioxidant, Wound healing, Anti-inflammatory, Biochemistry, Biotechnology, Microbiology, Plant sciences

## Abstract

Marine yeasts are underexplored resource for bioactive polysaccharides with potential pharmaceutical activities. In this study, five marine yeast isolates from mangrove shrubs were screened for β-glucan production. Isolate Y_4_ exhibited the highest β-glucan yield, reaching 17.11% of cell fresh weight- approximately 1.57-fold higher than *Saccharomyces cerevisiae*. Molecular identification revealed Y_4_ to be *Candida tropicalis* AUMC 15,533 through ITS rRNA sequencing and phylogenetic analysis. Structural characterization using ¹H NMR and FT-IR confirmed the presence of β-1,3 and β-1,6 glucan linkages. Optimization of β-glucan production via Plackett–Burman design identified glucose, yeast extract, shaking speed, and incubation time as critical factors, achieving a maximum yield of 0.42 g/100 mL. The extracted β-glucan demonstrated significant biological activities, including antioxidant capacity (IC₅₀= 18.31 µg/mL), wound healing efficacy (80.19% closure in 24 h), and anti-inflammatory effects, as evidenced by reduced expression of pro-inflammatory cytokines TNF-α (74.5% reduction) and IL-6 (80.8% reduction). Carboxymethylated β-glucan showed lower but still substantial activity across all assays. These findings highlight the superior biosynthetic capacity of marine *C. tropicalis* for β-glucan production and its potential therapeutic properties. This study underscores the importance of exploring marine microbial diversity for potential bioactive molecules with biomedical applications.

**Supplementary Information:**

The online version contains supplementary material available at 10.1038/s41598-026-42067-0.

## Introduction

Marine microorganisms, particularly those from extreme environments like the Red Sea, have evolved unique metabolic pathways to thrive under high salinity and temperature, making them exceptional sources of bioactive biomolecules^1^. Among these marine microorganisms, marine yeasts are notable producers of bioactive substances such as amino acids, glucans, enzymes, and vitamins, which hold significant promise for applications across the food, pharmaceutical, cosmetic, and chemical industries^2^^3^. Of particular interest is β-glucan which is considered as homo-polysaccharide that is made up of glucose units connected together, resulting in their significant β-(1→3) linkage backbone. The presence of hydroxyl groups facilitates hydrogen bonding with water, making β-glucans highly hydrophilic and capable of absorbing water in both soluble and insoluble forms^4^. β-glucans extraction methods differ according to their different sources. There are five basic extraction methods^5^ that can be used either solely or as a combination of two or more: Solvent Extraction, Enzymatic Extraction, Alkali Extraction, Hot Water Extraction and Ultrasound / Microwave-Assisted Extraction.

Yeast β-glucans are classified into α- and β-glucans, with β-1,3-glucan being the most abundant, representing 65–90% of the total β-glucan content^6^^7^. This polysaccharide is covalently linked to β-1,6-glucan, forming highly branched structures that contribute to the rigidity and functionality of the yeast cell wall^8^^9^. The physicochemical properties of β-glucan, such as its thickening, stabilizing, emulsifying, and gelling capabilities, make it a versatile compound with wide-ranging applications^10^.

Beyond its interesting physical properties, β-glucan exhibits remarkable biological activities, including wound healing promotion, collagen production enhancement, and protective effects against skin conditions such as wrinkles, acne, and eczema^11^. These properties have led to its incorporation into formulations for creams, ointments, suspensions, and powders, further highlighting its value in the cosmetic and pharmaceutical sectors^10^^11^. However, a significant knowledge gap persists in the biotechnological exploitation of marine-derived yeasts^12^^13^. Furthermore, the extraction and purification of β-glucan require optimization to ensure cost-effectiveness and scalability while preserving its native structure and functional properties^14^^15^.

While terrestrial *Saccharomyces cerevisiae* is the conventional source for β-glucan, the structural and functional potential of β-glucans from extremophilic strains, such as those found in the Red Sea, remains largely unexplored. Specifically, there is a lack of evidence regarding how the unique environmental pressures of the Red Sea influence the molecular architecture and, consequently, the antioxidant and anti-inflammatory efficacy of β-glucan derived from *Candida tropicalis*.

To address these gaps, the current study focuses on the isolation and identification of a β-glucan-producing *Candida tropicalis* strain from the Red Sea. We aim to optimize its production and provide a comprehensive characterization of its structural properties using NMR and FT-IR^15,16^. Furthermore, this research moves beyond basic characterization to specifically evaluate the strain’s potential in wound healing, anti-inflammatory, and antioxidant applications. By bridging the gap between marine microbial diversity and pharmaceutical application, this work seeks to establish Red Sea-derived β-glucan as a high-performance alternative to traditional terrestrial sources.

## Materials and methods

### Media

**Sabouraud agar medium** (Difo™): was prepared according to the manufacturer instructions with 50% seawater and Amoxicillin 100 mg/L was used as a broad- spectrum antibiotic in the initial steps of isolation. This medium was used for yeast isolation, purification, and preservation.

**YPD medium** (g/L): glucose 20, yeast extract 10, peptone 20 was used for β-glucan production. Constituents were dissolved in 100 mL distilled water and pH was adjusted to 5 using NaOH and/or HCl.

**BD Difco™ Czapek solution agar**: was prepared according to the manufacturer instructions and used for yeast culture preparation for subsequent molecular identification.

Liquid media were prepared from the same constituents without adding agar-agar. All media were sterilized by autoclaving at 120 ℃ for 20 min.

### Chemicals

All chemicals and solvents were obtained from Difco™ Company. Enzymes, primers and Patho-gene-spin DNA/RNA extraction kit were obtained from Intron Biotechnology Company in Korea.

### Cell lines

Epithelial-like cells (WISH, ATCC: CCL-25) are human epithelial cells contains HeLa marker chromosomes. The cells were obtained from Nawah Scientific, Egypt. The guidelines of animal cells culture described by the American Type Culture Collection (ATCC) were followed. The maintenance of the cultures was performed in high-glucose DMEM (Dulbecco’s modified Eagle’s medium), fortified with glucose (4.5 g/L), L-glutamine (4 mM) and FBS (10%) (Lonza, UK).

### Isolation of yeasts

For marine yeasts isolation, samples were collected from aerial roots around mangrove shrubs (*Avicennia marina)*, Hidden Bay, Ras Mohammed Protectorate (Supplementary Fig. S1)., Sinai, Egypt (27.7383° N, 34.2427° E). The host plant, *Avicennia marina*, is classified as ‘Least Concern’ on the IUCN Red List of Threatened Species. It is a widely distributed species and is neither endangered nor near threatened. The plant was previously identified by Edward Vanden Berghe, with voucher specimens deposited at the Royal Botanic Gardens, Kew, Surrey, UK (K000235040) and the Natural History Museum, London (BM000950260). Root samples collection was managed under the protectorate’s regulations involving a minimal number of small root samples which were non-destructive and did not harm the plant. Prior to collection, formal permission was obtained through an official request submitted by the Faculty of Science, Alexandria University, to the Sinai Protectorates Administration. The sampling was carried out under the supervision of an official guide assigned by the protectorate to ensure full compliance with local environmental regulations. Parts of about 10 cm from the plant aerial roots were cut and shaken vigorously in 10 mL of sterile 0.9% saline. Dilutions of 10^− 1^, 10^− 2^, 10^− 3^, 10^− 4^ and 10^− 5^ were prepared and 1 mL of each dilution was inoculated into Sabouraud Dextrose Agar (SDA) medium containing 1% amoxicillin. All plates were incubated at 25 ℃ for 5 days with daily observation for yeast colony development^17^. *Saccharomyces cerevisiae* was isolated from commercial dry baker’s yeast and cultured on the same medium.

### β-glucan extraction and purification

As glucan is a cell wall component of yeast, it was aimed to enhance yeast biomass production. One mL of a freshly prepared yeast cell suspension, where cell count was 10^6^ CFU/mL, was used to inoculate 100 mL of sterile YPD medium in a 250 mL Erlenmeyer flask. The flasks were incubated in a shaking incubator at 200 rpm and 25 °C for five days. The biomass was harvested by centrifugation at 4000 rpm for 20 min. The supernatant was discarded, and the cells were washed three times with 0.9% sterile saline solution before being subjected to glucan extraction.

About 1 g fresh yeast cells was boiled in 150 mL of 0.1 N NaOH for 15 min. at 100 °C with stirring. The residue was collected by centrifugation at 4000 rpm for 20 min. and washed with 50 mL distilled water. The residue was then resuspended in distilled water where pH was adjusted to 7 ± 0.1. The residue was subjected to boiling in 150 mL of 0.1 N HCl for 15 min in clean flasks to avoid any contamination with any remaining base. The residue was washed again with 50 mL distilled water. After alkali and acid treatment, the residue should be free of cellular proteins and other polysaccharides. To get rid of lipids, the residue was suspended in 50 mL ethanol and left overnight, then the suspension was boiled at 80 °C for 15 min and centrifuged at 4000 rpm for 30 min. The residue was washed 3 times in 50 mL distilled water, boiled for 15 min then centrifuged^14^^15^. The residue was resuspended in least amount of distilled water in pre-weighed 50 mL vial to calculate the yield percentage. Residue were stored either frozen or lyophilized.

The extracted β-glucan was quantified, and the yield was expressed as a percentage relative to that obtained from the reference strain *Saccharomyces cerevisiae*. This comparison allowed the evaluation of the production capacity of each marine isolate to select the most potent isolate.

The conversion of β-glucan from insoluble form to soluble form was carried out by the process of carboxymethylation (CM) through a two-step alkalization and etherification. Briefly, 2 g of β-glucan was left to swell in 50 mL of 80% ethanol for one hour. Then 4 mL of 50% NaOH and 15 mL of 4 M chloroacetic acid were added gradually into the mixture with stirring for two hours at 50℃. The second step of alkalization was done by adding 3 mL of 50% NaOH with stirring for 3 h at 50℃. A neutralization step was carried out by using 1 M acetic acid or 1 M NaOH. Then the resulting mixture was filtered and washed with 80% methanol followed by absolute methanol then dried at 50℃ until reaching constant weight. Finally the dry carboxymethylated CM-β-glucan was stored in a desiccator for further analysis^18^.

### Molecular identification of the selected isolate

Strain Y_4_ was cultured on Czapek`s agar (CZA) medium for 7 days at 28°C^19^. Fresh culture was sent to the Molecular Biology Research Unit, Assiut University for DNA extraction using Patho-gene-spin DNA/RNA extraction kit provided by Intron Biotechnology Company, Korea. Samples of yeast DNA were shipped to SolGent Company, Daejeon, South Korea for polymerase chain reaction (PCR) and sequencing of the internal transcribed spacer (ITS) region of the yeast rDNA. PCR was performed using two universal primers namely ITS1 (forward) and ITS4 (reverse). The primers have the following sequences: ITS1 (5’ - TCC GTA GGT GAA CCT GCG G − 3’) and ITS4 (5’- TCC TCC GCT TAT TGA TAT GC -3’). Purified PCR product were sequenced with the same primers with the incorporation of ddNTPs in the reaction mixture^20^. The obtained sequences were analyzed using Basic Local Alignment Search Tool (BLAST) from the National Center of Biotechnology Information (NCBI) website. Phylogenetic analysis of the sequences was performed using MegAlign (DNA Star) software 5.05.

### Growth curve analysis of isolate Y4

The growth curve analysis of isolate Y4 was performed to determine the growth phases according to time to detect the late exponential to the stationary phase, where the yeast cell walls become thicker and produce higher yield of β-glucan^21,22^. YPD medium was prepared for 2 flasks: control and test. The control medium was used as standard to measure the OD of the test culture.

### β-glucan characterization

#### FT-IR analysis

The FT-IR spectra of the extracted β- Glucan was measured by KBr method in the central lab of Faculty of science– Alexandria University with (Bruker Tensor 37 FT-IR, Bruker, Germany). A total of 5 mg of powdered extract was blended with 200 mg of KBr, then compressed into pellets. The resulting KBr pellets were examined over the spectral range of 4000 to 500 cm⁻¹ at 27 °C, using 50 scans and a resolution of 4 cm⁻¹^23^.

#### NMR analysis

The^1^ H NMR spectra were obtained from dissolving the extract in DMSO then in D_2_O by Mercury-300BB “NMR300”, operating at 300.0687872 MHz, relax delay 6.000 s, pulse 45.0-degree, acquisition time 4.000 s and width 6600.7 Hz. Interpretation of spectra were performed based on peaks observed at different frequencies (ppm)^24^.

### Optimization of yeast cell growth

To study the key factors influencing β-glucan yield, seven independent variables were selected for evaluation: glucose concentration (g/L), yeast extract (g/L), peptone (g/L), initial pH, agitation speed (rpm), seawater percentage (%), and incubation time (day). Each factor was tested at three levels: low, basal, and high (supplementary table S2). A Plackett–Burman’s design (supplementary table S3) was applied to assess the relative influence of those independent factors on β-glucan yield (g/100 mL). Experimental data were compiled and analyzed using Microsoft Excel, and regression analysis was employed to identify the factors exerting the most significant main effects. After the optimization was performed, 3 trials confirmation experiment was conducted to check the accuracy of results. First trial was selected to be the optimized trial with best result in the previous step. While the second trial was prepared to be the exact opposite of the optimized trial and named “anti-optimized”. The third trial was prepared as basal trial of the optimization step.

### Pharmaceutical applications of β-glucan

#### Antioxidant activity

Yeast β-glucan and CM-β-glucan from *C. tropicalis* AUMC 15533were tested for antioxidant activity at Pharmaceutical and Fermentation Industrial Center (General Authority of City of Scientific Research and Technological Application, in Burg ElArab). The free radical scavenging activity was measured by the DPPH method^25^. A solution of 0.2 mM DPPH in methanol (0.0078 g/100 mL) was prepared and 1 mL of this radical solution was added to 1 mL of extract or ascorbic acid at different concentrations (1:1 v/v). The mixture was incubated for 30 min in the dark at room temperature and then the absorbance was measured at 517 nm using a spectrophotometer. Ascorbic acid solutions as standards in the concentration range of (5–200 µg/mL) were used to establish a calibration curve. DPPH radical scavenging activity was expressed as mg ascorbic acid equivalent (AAE)/g dried sample. The percentage DPPH radical-scavenging activity was calculated using Eq. (1):

1 $$\:\mathbf{D}\mathbf{P}\mathbf{P}\mathbf{H}\:\mathbf{r}\mathbf{a}\mathbf{d}\mathbf{i}\mathbf{c}\mathbf{a}\mathbf{l}\:\mathbf{s}\mathbf{c}\mathbf{a}\mathbf{v}\mathbf{e}\mathbf{n}\mathbf{g}\mathbf{i}\mathbf{n}\mathbf{g}\:\mathbf{a}\mathbf{c}\mathbf{t}\mathbf{i}\mathbf{v}\mathbf{i}\mathbf{t}\mathbf{y}\:\left(\mathbf{\%}\:\mathbf{i}\mathbf{n}\mathbf{h}\mathbf{i}\mathbf{b}\mathbf{i}\mathbf{t}\mathbf{i}\mathbf{o}\mathbf{n}\right)=\frac{({\boldsymbol{A}\boldsymbol{b}\boldsymbol{s}}_{\boldsymbol{C}\boldsymbol{o}\boldsymbol{n}\boldsymbol{t}\boldsymbol{r}\boldsymbol{o}\boldsymbol{l}}-{\boldsymbol{A}\boldsymbol{b}\boldsymbol{s}}_{\boldsymbol{t}\boldsymbol{e}\boldsymbol{s}\boldsymbol{t}})}{{\boldsymbol{A}\boldsymbol{b}\boldsymbol{s}}_{\boldsymbol{C}\boldsymbol{o}\boldsymbol{n}\boldsymbol{t}\boldsymbol{r}\boldsymbol{o}\boldsymbol{l}}}\times\:100 $$

#### Anti-inflammatory effect

To differentiate between cytotoxic effects and anti-inflammatory activity, WISH cells were first stimulated with lipopolysaccharide (LPS) at a concentration of 20 µg/mL to induce an inflammatory response. Subsequently, the cells were treated with piroxicam (10 µg/mL), β-glucan, and carboxymethylated β-glucan (CM-β-glucan), each applied at one-tenth of their respective IC₅₀ values. IC_50_ is the concentration at which the product demonstrates half of its maximal inhibitory effect. The concentrations prepared to test cell viability for β-glucan and CM-β-glucan were 2500, 1250, 625, 312.5, 156.25, 78.125 µg/mL for each.

The anti-inflammatory potential of β-glucan and CM-β-glucan (the Carboxymethylated form) was assessed using WISH human epithelial, (ATCC: CCL-25). Initially, their cytotoxicity was evaluated to determine safe concentrations for further testing, using lipopolysaccharide (LPS)- a well-known inducer of inflammation- and piroxicam, a standard anti-inflammatory agent, for comparison. The MTT assay was employed to distinguish viable, metabolically active cells from non-viable ones. For the assay, 100 µL of DMEM culture medium (optical density 0.05 U) containing 5 × 10⁴ WISH cells were seeded into each well of a 96-well plate^26^. After cell attachment, 50 µL of LPS was added to all wells except those designated as untreated controls, to induce an inflammatory response. The plate was incubated at 37 °C in a humidified CO₂ incubator (5% CO₂, 90% RH) for 24 h. Supernatants were gently removed and 750 µL of a 1:10 dilution of the IC₅₀ of each test sample or piroxicam was added. LPS and piroxicam were used at final concentrations of 20 µg/mL and 10 µg/mL, respectively. The plate was then incubated for an additional 48 h under the same environmental conditions. At the end of this period, the MTT assay was performed to assess cell viability which was calculated using Eq. (2):

2 $$ \:\mathbf{C}\mathbf{e}\mathbf{l}\mathbf{l}\:\mathbf{V}\mathbf{i}\mathbf{a}\mathbf{b}\mathbf{i}\mathbf{l}\mathbf{i}\mathbf{t}\mathbf{y}\:\left(\mathbf{\%}\right)=\left(\frac{{\boldsymbol{A}}_{\boldsymbol{t}}-\:{\boldsymbol{A}}_{\boldsymbol{b}}}{{\boldsymbol{A}}_{\boldsymbol{c}}-{\boldsymbol{A}}_{\boldsymbol{b}}}\right)\mathbf{*}100 $$

Proliferation was analyzed by comparing the viability of untreated control cells to those treated with LPS alone, piroxicam, or β-glucan and CM-β-glucan samples.

To gain deeper insight into the anti-inflammatory mechanism of β-glucan and CM-β-glucan, the expression levels of four major pro-inflammatory cytokines- TNF-α and IL-6- were quantified in LPS-stimulated WISH cells. These cytokines are central to the inflammatory response, contributing to vasodilation, edema, and the development of autoimmune and chronic inflammatory conditions.

Total RNA was extracted from cells treated with β-glucan, CM-β-glucan, and piroxicam, alongside LPS-stimulated (negative control) and untreated (positive control) cells. RNA extraction was conducted using a commercial kit (iNtRON Biotechnology, Korea), following the manufacturer’s guidelines. The isolated RNA was then reverse transcribed into cDNA using the SensiFAST cDNA synthesis kit (Bioline, London, UK). Quantitative PCR (qPCR) was performed to measure gene expression levels, using GAPDH as the internal housekeeping gene and specific primers (Supplementary Table S4) for each target cytokine.

For each qPCR reaction, 12.5 µL of SensiFAST SYBR Green Master Mix was combined with 1 µL of cDNA, 0.5 µL each of forward and reverse primers (10 pmol/µL), and nuclease-free water to a final volume of 20 µL. Amplification was carried out using a CFX96™ Real-Time PCR System (BIO-RAD, USA) under the following conditions: initial denaturation at 95 °C for 10 min, followed by 40 cycles of denaturation at 95 °C for 15 s, annealing at 60 °C for 30 s, and extension at 72 °C for 30 s. Gene expression levels were quantified using the 2^−ΔΔCt^ method, with normalization to GAPDH^27^.

The Stimulation Index (SI) was calculated by dividing the mean absorbance of LPS-stimulated cells (with or without treatment) by the absorbance of untreated control cells. This value indicates the extent to which each treatment mitigates LPS-induced cellular effects.

The Effective anti-inflammatory concentration (EAIC) refers to the concentration at which each tested extract effectively suppresses the overexpression of inflammatory cytokines, restoring cellular behavior to levels observed in non-inflamed (control) conditions. This reflects the extract’s capacity to attenuate inflammatory responses and normalize aberrant cell proliferation caused by LPS, primarily by downregulating the expression of TNF-α and IL-6.

To assess the safety margin and therapeutic efficiency of β-glucan and CM-β-glucan, the Therapeutic Index (TI) was calculated Eq. (3):

3 $${\text{TI = (EAIC / IC}}_{{{\mathrm{50}}}} {\mathrm{)}} \times {\mathrm{100}}$$

where lower TI values indicate a wider safety margin.

#### Wound healing effect

Impaired skin regeneration involves complex biological mechanisms, particularly the process of re-epithelialization. This process relies on the migration of epithelial cells from the wound margins to restore the epidermal layer^28^. Wound assay was performed for the extracted β-glucan and CM-β-glucan on WISH human epithelial (ATCC: CCL-25). The in vitro scratch assay is an easy, well-developed method to measure migration in vitro. The main steps involve coating the cell culture dishes, passaging the cells in culture and creating a scratch in a cell monolayer, then capturing the images at the beginning and after intervals of 24 h of cell migration until wound closer^29^. This experiment was carried out at Faculty of Science, Alexandria University and wound closure percentage was measured using Eq. (4):

4 $$ \:\mathbf{W}\mathbf{o}\mathbf{u}\mathbf{n}\mathbf{d}\:\mathbf{c}\mathbf{l}\mathbf{o}\mathbf{s}\mathbf{u}\mathbf{r}\mathbf{e}\:\left(\mathbf{\%}\right)=\frac{({\mathbf{W}\mathbf{o}\mathbf{u}\mathbf{n}\mathbf{d}\:\mathbf{L}\mathbf{e}\mathbf{n}\mathbf{g}\mathbf{t}\mathbf{h}}_{0\:\mathbf{H}\mathbf{o}\mathbf{u}\mathbf{r}}-{\mathbf{W}\mathbf{o}\mathbf{u}\mathbf{n}\mathbf{d}\:\mathbf{L}\mathbf{e}\mathbf{n}\mathbf{g}\mathbf{t}\mathbf{h}}_{24\:\mathbf{H}\mathbf{o}\mathbf{u}\mathbf{r}\mathbf{s}})}{{\mathbf{W}\mathbf{o}\mathbf{u}\mathbf{n}\mathbf{d}\:\mathbf{L}\mathbf{e}\mathbf{n}\mathbf{g}\mathbf{h}\mathbf{t}}_{0\:\mathbf{H}\mathbf{o}\mathbf{u}\mathbf{r}}}\times\:100 $$

##### Coating the cell culture dishes

60 mm dishes were coated with extracellular matrix ECM substrates and incubated overnight at 4 °C without shaking or rotation. Then, the unbound ECM substrate was removed, and the coated dishes were blocked with 3 mL of 2 mg/mL bovine serum albumin for 1 h at 37 °C.

##### Passaging the cell in culture

The sub confluent growing cells were resuspended in a tissue culture dish by washing the cells twice with PBS, adding versine containing trypsin, and then mixing the cells with medium containing serum. Gently rock the dish to disperse the cells equally. Take an aliquot from the cells suspension and place them onto 60 mm dish to create a confluent monolayer. Incubates the dishes for 6 h at 37 °C allowing the cells to adhere and spread on the substrate completely.

##### Scratch assay

The cell monolayer was scraped in a straight line with a µ200 pipet tip. The debris were removed, and the edges were smoothed by washing with 1 mL of the growing medium, then replaced with 5 mL of medium specific for the in vitro scratch assay. The dishes were incubated at 37 °C for 24 h for the time frame of examination. The software used for image analysis was (ZOE fluorescent cell imager).

## Results and discussion

### Isolation of yeast

Five marine yeast isolates were obtained (Y_1_, Y_2_, Y_3_, Y_4_, Y_5_). *S. cerevisiae* was used as reference for β-glucan production. The colony morphology and cells shape are shown in Supplementary Fig. S8. Isolated yeasts have round creamy raised colonies varying in size. Marine yeasts are reported to be isolated from mangrove shrubs. Mangroves are expected to be rich in microbial diversity attached to their aerial roots^30^. Along the Egyptian Red Sea coast, mangrove vegetation consists of a single species, *Avicennia marina*, covering about 525 hectares. The only exception occurs in a few areas close to the Egyptian Sudanese border, where *Rhizophora mucronata* grows alongside *A. marina*^31^.

### Screening for β-glucan production

The five marine yeast isolates were screened for β-glucan yields in comparison to *S. cerevisiae* (Supplementary Fig. S9). Isolate Y_4_ showed the highest β-glucan content, reaching 17.11% of cell fresh weight. This yield corresponds to approximately 1.57-fold higher than that observed in *S. cerevisiae*, indicating its superior biosynthetic capacity. The remaining isolates showed lower yields. Accordingly, Y_4_ was selected for further studies.

*S. cerevisiae* was found to contain *10.11*% β-glucan relative to total cell biomass. This value is consistent with recent reports^32^. Such agreement confirms the reliability of the extraction and quantification method used in this work. Notably, while *S. cerevisiae* is widely recognized as a conventional source of β-glucan, our findings demonstrate that some marine isolates, particularly Y_4_, can surpass its yield, producing up to *17.11*% of fresh cell weight. This enhanced production capacity suggests that marine yeasts, adapted to unique environmental conditions such as high salinity and nutrient fluctuations, may possess more efficient biosynthetic pathways for β-glucan synthesis compared to terrestrial strains^33^. Sukumaran et al.^34^ documented that the glucan content of marine yeast ranged from *8.1% to 12.4%*, expressed relative to the original biomass, with the lowest values recorded in *Candida tropicalis* and the highest in two strains of *Debaryomyces hansenii*. The fact that Y_4_ surpassed this reported range highlights its exceptional biosynthetic capacity and potential as a superior marine source of β-glucan.

### Molecular identification of isolate Y4

The internal transcribed spacer (ITS) regions of the 18 S rRNA gene of isolate Y_4_ were successfully amplified using the universal primers ITS1 and ITS4, yielding an amplicon of 438 bp. The purified PCR product was sequenced, and the resulting sequence was analyzed through BLAST alignment against the NCBI GenBank database. The analysis revealed 99.7% similarity with *Candida tropicalis* sequences. Phylogenetic analysis was performed using MegAlign (DNA Star) software, and the resulting tree confirmed the close clustering of the Y4 isolate with *C. tropicalis* reference strains, showing 100% sequence identity and coverage. *Rhodotorula mucilaginosa* was used as an outgroup, clearly separating the Y_4_ isolate within *Candida tropicalis* clade (Supplementary Fig. S10). Accordingly, the isolate was identified as *Candida tropicalis* and deposited in Assiut University Mycological Center (AUMC), Assiut, Egypt, under the accession code AUMC 15,533. The ITS sequence was deposited in the GenBank under the accession number ON614153. *Candida tropicalis* is a well-documented yeast species commonly isolated from diverse environments particularly mangroves and has been reported as a source of bioactive metabolites, including β-glucans with immunomodulatory and therapeutic potential^35^^36^.

### Growth curve of *Candida tropicalis* AUMC15533

**Fig. 1:**
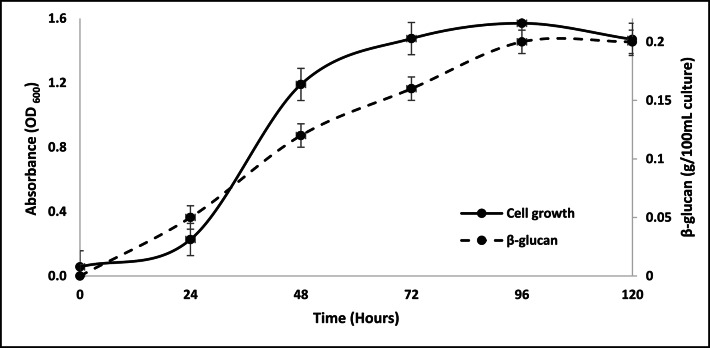
Growth curve of *Candida tropicalis* AUMC15533 showing β-glucan content.

The growth curve in (Fig. 1) showed the exponential phases of *Candida tropicalis* AUMC15533 in the range 24–48 h, while stationary phase was terminated at nearly 120 h where the death phase was about to start. In logarithmic phase, yeast cells were growing, dividing and building their cell wall components including β-glucan. While in stationary phase, the cell growth speed decreased due to nutrient depletion. During this phase, the cell wall composition can be changed and the cell biomass is maximized, which is generally the favorable time for high yield of β-glucan production^37^.

### Chemical characterization of β-glucan

#### FT-IR analysis

**Fig. 2:**
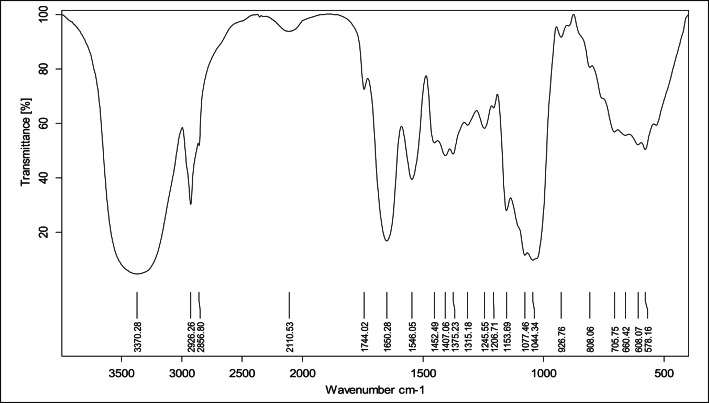
FT-IR analysis of the extract obtained from *C. tropicalis* AUMC 15,533.

FTIR analysis of β-glucan extract from *C. tropicalis* AUMC 15,533 (Fig. 2) revealed characteristic absorption bands confirming the presence of polysaccharide structures. A broad and intense band in the 3100–3600 cm⁻¹ region corresponded to axial O–H stretching vibrations, indicative of abundant hydroxyl groups typical of β-D-glucans. A distinct absorption near 2920–2926 cm⁻¹ was attributed to C–H stretching vibrations of carbohydrate backbones. In the mid-infrared region, characteristic polysaccharide peaks were observed, including bands at 1407–1452 cm⁻¹ corresponding to C–H bending, ~ 1153 cm⁻¹ assigned to C–O–C bridge stretching, and ~ 1077 cm⁻¹ reflecting C–O stretching within the glucose ring. Peaks at 1044 cm⁻¹ and 1077 cm⁻¹ were further associated with the stretching vibrations of C–O and C–C bonds, representing (1→3)-β-D-glucan and (1→6)-β-D-glucan linkages, respectively. Finally, all spectra displayed a band near 800 cm⁻¹, which is a well-recognized signature vibration for β-glycosidic linkages, particularly those of β-1,3 and β-1,6 configurations. Together, these consistent absorption patterns strongly confirm the successful extraction and structural integrity of β-glucans from the chosen isolate. 

Collectively, the FTIR spectra from *C. tropicalis* AUMC 15,533 exhibited key absorption bands consistent with the molecular fingerprint of β-glucans. The prominent band at ~ 890 cm⁻¹ is widely recognized as the indicator of β-anomeric linkages, especially β-1,3 or β-1,6 glycosidic bonds. This region has been documented as a reliable diagnostic feature for β-glucans in multiple studies^34^. Furthermore, the presence of broad O–H stretching vibrations around 3400 cm⁻¹ was reported by^38^ confirming the highly hydroxylated nature of β-glucan polysaccharides. In addition, peaks near 1150 and 1070 cm⁻¹ correspond to glycosidic linkages and ring vibrations within glucose monomers, which aligns with findings by^39^ who reported that those peaks are characteristic of fungal β-glucan. This consistency with previously reported findings in the literature strongly reinforces the conclusion that the extracted compounds are indeed β-glucans. Similar data were also obtained for β-glucan produced by *Pediococcus parvulus*^40^.

#### NMR analysis

**Fig. 3:**
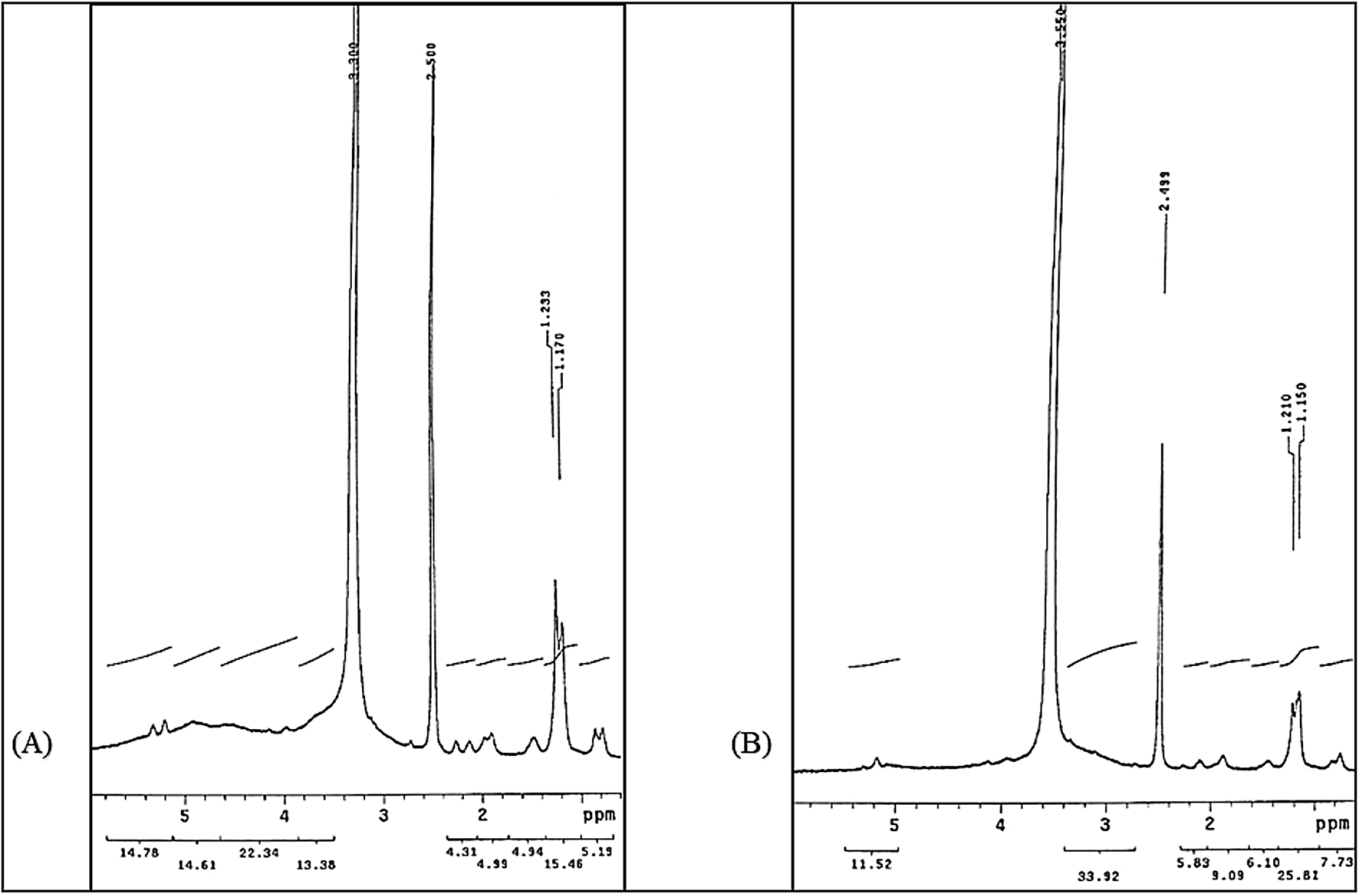
Proton NMR analysis of the extract obtained from *C. tropicalis* AUMC 15,533 dissolved in (**A**) DMSO (**B**) D_2_O.

¹H NMR analysis was performed to confirm the presence and purity of β-glucan in the extracted samples. As shown in Fig. 3, the spectra exhibited characteristic resonances of yeast-derived β-glucans. In the anomeric proton region, a distinct signal at ~ 5.3 ppm corresponded to α-glycosidic linkages, while signals observed at δ 4.4–4.8 ppm were typical of β-anomeric protons, confirming the predominance of β-1,3 and β-1,6 linkages^41^. The absence of additional peaks in the α-anomeric region (5.0–5.5 ppm) further supports the β-configuration of the glucose units. Moving downfield, the δ 3.2–4.0 ppm range showed multiple patterns attributed to ring protons (H-2 to H-6) of the glucopyranose units, consistent with the polysaccharide backbone. Together, these chemical shifts form the fingerprint region characteristic of β-glucans. Importantly, no unexpected sidechain signals were detected apart from hydroxyl-associated resonances, indicating the high purity of the extracted glucan. The combined spectral features confirm the β-configuration and structural integrity of the glucans obtained from *Candida tropicalis* AUMC 15,533, in agreement with previously reported yeast β-glucan spectra^24^.

### Optimization of β-glucan production

**Fig. 4:**
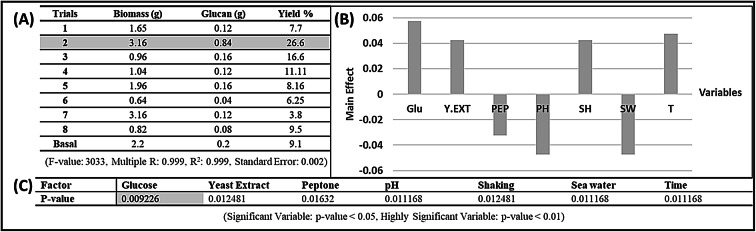
(**A**) Optimization for β-glucan production by *C. tropicalis* AUMC 15,533. (**B**) The Main Effect chart for statistical analysis of optimization of β-glucan production from *Candida tropicalis* AUMC 15,533 (**C**) P-value of the variables used in Plackett-Burman Design.

The Plackett–Burman design was applied to evaluate the effects of seven independent variables on the dry weight yield of β-glucan from *C. tropicalis* AUMC15533 (g/100 mL). The average outcomes of nine experimental trials are presented in Fig. 4-A, with Trial 2 yielding the highest β-glucan production (0.84 g/100 mL). Data in Supplementary Table S5 presents the responses of the verification experiment. It showed that the optimized trial has the highest yield percent. The ANOVA analysis (Supplementary Table S6) demonstrated statistical significance (F = 3033, *p* = 0.013) and a high coefficient of determination (R² = 0.999), confirming that 99.9% of the variability was explained by the model and establishing its robustness in predicting β-glucan yield. The main effects analysis (Fig. 4-B) revealed that glucose, yeast extract, shaking speed (SH), and incubation time (T) positively influenced β-glucan production, whereas peptone, pH, and seawater percentage had negative effects. Among these, glucose was identified as the most critical factor, as higher glucose levels were strongly associated with increased glucan yield. Similarly, increasing yeast extract, SH, and T contributed positively, while elevated peptone, pH, and seawater salinity reduced yield.

These findings align with previous reports demonstrating that glucose concentration, yeast extract, pH, temperature, and culture medium composition significantly influence β-glucan production in *Saccharomyces cerevisiae*^42^. Similarly, our observation of reduced yields under elevated salinity is consistent with earlier studies, such as those on VIN 13 *S. cerevisiae*, which reported that increasing salt concentrations suppressed β-glucan synthesis^43^. The salinity of the water samples from which the yeast was isolated was measured at 2.76%, which is lower than the average Red Sea salinity of 4.1%. This difference may explain why *C. tropicalis* isolated in the present study exhibited optimal growth under low-salinity conditions. The reduced salinity could be linked to the influence of desalination plant discharge along the northern coast of the Red Sea in Egypt^44^. Furthermore, it is likely that *C. tropicalis* was not exposed to salinity stress in its natural niche on mangrove aerial roots, as recent studies have shown that these roots possess an ultrafiltration mechanism, functioning like reverse osmosis, that separates salts from seawaters^45^. Together, these results emphasize that both nutritional and abiotic factors play critical roles in regulating β-glucan biosynthesis, and their careful optimization is essential for maximizing yield. Additionally, numerous studies have reported that culture incubation period plays a crucial role in bioactive compounds production as reported by Gunasekran and Poorniammal 2008^46^. The positive main effect of shacking rpm emphasizes the complex influence of aeration on fungal metabolic activity which is in agreement with findings from various studies. For example Chysirichote et al.^47^ have reported that higher aeration rates promoted increased fungal growth.

### Pharmaceutical applications of β-glucan

#### Antioxidant activity

**Fig. 5:**
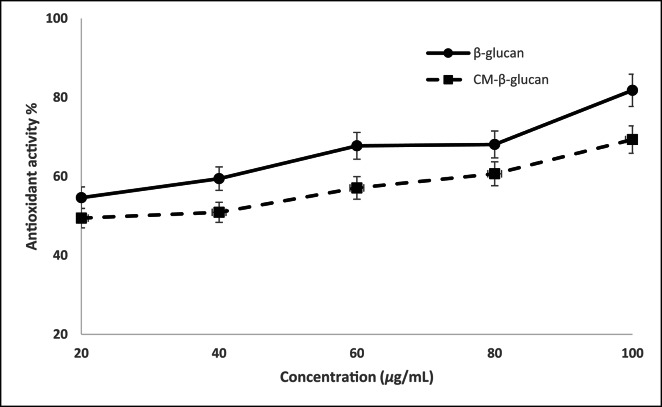
The relationship between concentration of β-glucan or CM-β-glucan against the corresponding antioxidant activity using DPPH method.

To assess the antioxidant activity of β-glucan produced by *C. tropicalis* AUMC 15,533, DPPH was measured in its presence in comparison to ascorbic acid. Data in Supplementary Table S7 showed that β-glucan reduced DPPH by 50% at concentration of 18.31 µg/mL while reduction of the same percentage required 39.29 µg/mL of its carboxymethylated form. The maximum antioxidant activity was observed at a concentration of 100 µg/mL, reaching 81.80% for extracted β-glucan and 69.33% for its carboxymethylated form (Fig. 5).

Those results demonstrated significant radical scavenging potential for both samples. The IC₅₀ values were determined to be 18.31 µg/mL for β-glucan and 39.29 µg/mL for CM-β-glucan (Supplementary Table S6), indicating a higher antioxidant potency of β-glucan compared to its carboxymethylated derivative. These values reflect the concentration required to neutralize 50% of the DPPH radicals and serve as a measure of antioxidant strength- the lower the IC₅₀, the higher the scavenging ability.

At a concentration of 100 µg/mL, which is often used as a benchmark in antioxidant studies, the maximum DPPH radical inhibition achieved was 81.80% for β-glucan and 69.33% for CM-β-glucan (Fig. 4). These results are noteworthy when compared to those reported by Abdel Ghany et al. (2016); Schiavone et al. (2014)^16^^40^, who found that the highest antioxidant activity of β-glucan reached only 60% at the same concentration. Moreover, despite using lower concentrations of β-glucan than those reported by Kath and Kulicke (1999) ; Zhang et al. (2022)^48^^49^, our study achieved comparable levels of antioxidant activity, highlighting the efficiency of both the extracted and carboxymethylated forms. Our findings thus demonstrate significantly enhanced antioxidant performance, particularly for the native β-glucan extract.

The promising antioxidant capacity observed in this study may be attributed to the extraction method, purity, molecular weight, or branching structure of the β-glucan used. The relatively lower activity of CM-β-glucan compared to the native form might be due to structural modifications introduced during carboxymethylation, potentially affecting the number of available hydroxyl groups responsible for radical scavenging. Overall, the high antioxidant activity demonstrated- especially by β-glucan- supports its potential application as a natural antioxidant agent.

The highest concentration used in this work is lower than that of recent study and have similar antioxidant effect^49^. In this study, the native β-glucan exhibited superior antioxidant activity compared to its carboxymethylated derivative (CM-β-glucan). This observed decrease in radical scavenging capacity following modification can be attributed to the substitution of native hydroxyl (-OH) groups with carboxymethyl groups. The antioxidant mechanism of polysaccharides is heavily dependent on the availability of free hydroxyl groups, which act as hydrogen donors to neutralize reactive oxygen species (ROS)^50^. By substituting these active hydrogen-donating sites, the carboxymethylation process likely reduced the density of functional groups available for radical stabilization^51^. Furthermore, the introduction of bulky carboxymethyl groups may induce steric hindrance, restricting the conformational flexibility of the polysaccharide chain and preventing the remaining active sites from effectively interacting with the radicals^52^. Similar trends have been reported in other polysaccharide studies, where excessive substitution was found to diminish rather than enhance antioxidant potency despite improving solubility^4^.

#### Anti-inflammatory effect

**Fig. 6:**
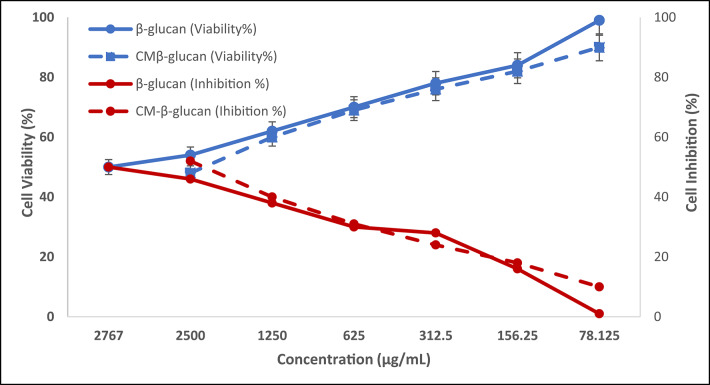
Cell viability of MTT assay when applied on WISH human cells to determine cytotoxicity of extracted β-glucan and CM-β-glucan.

Cell viability was assessed using the MTT assay (Fig. 6). This assay also enabled the determination of IC₅₀ values for both β-glucan and CM-β-glucan, which were calculated to be 2767 µg/mL and 2281 µg/mL, respectively.

**Fig. 7:**
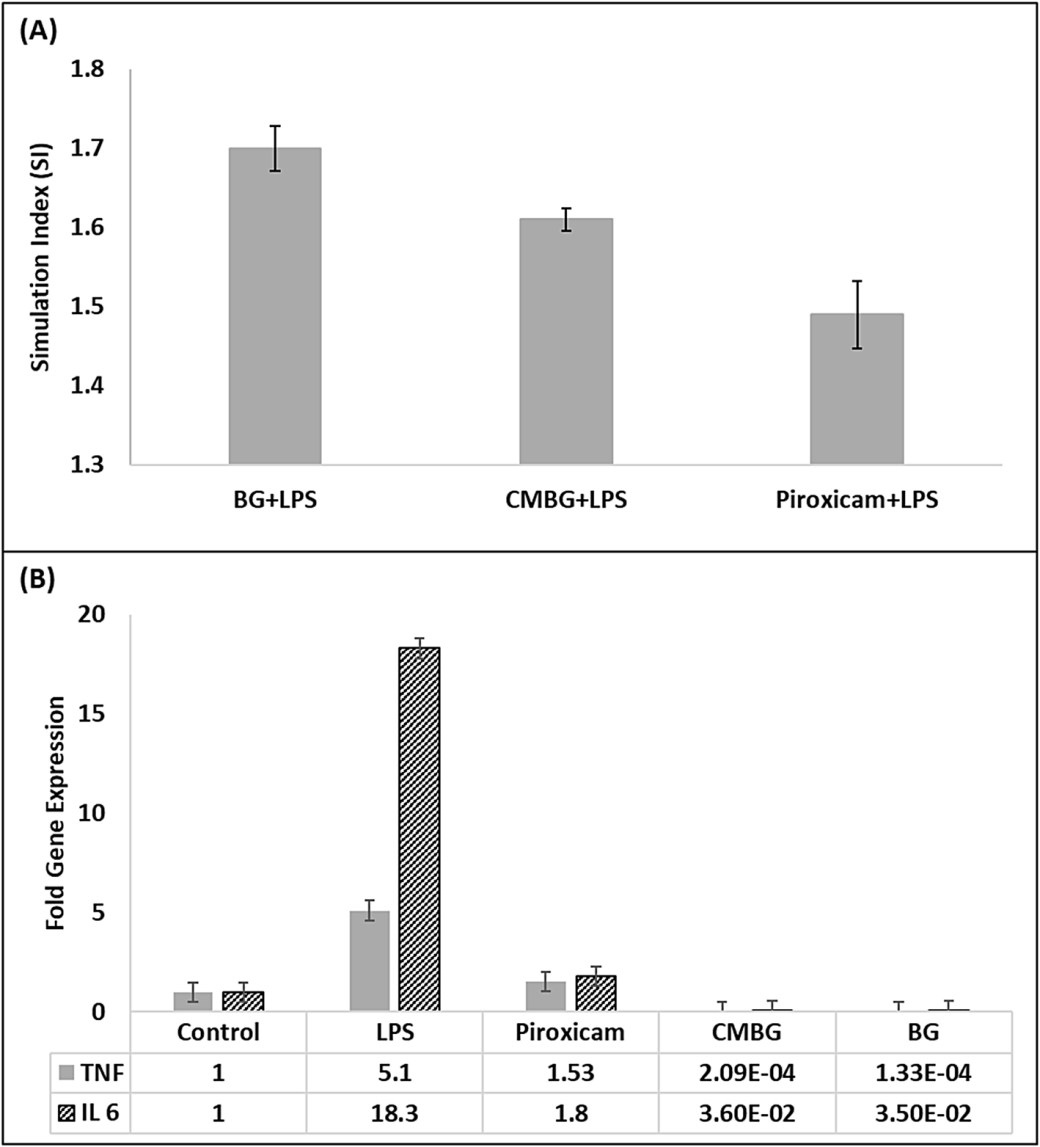
(**A**) Stimulation Index of each compound compared to Piroxicam when added to WISH human cells treated with LPS. (**B**) Fold gene expression results for anti-inflammatory effect of extracted β-glucan and CM-β-glucan.

The stimulation index (SI) was employed to evaluate the capacity of each treatment to mitigate LPS-induced cytotoxicity. Piroxicam treatment yielded an SI of 1.49 (Fig. 7-A). Notably, both β-glucan and CM-β-glucan demonstrated superior performance, achieving SI values of 1.70 and 1.61, respectively, thereby surpassing the reference drug piroxicam. The Effective Anti-inflammatory concentration (EAIC)- defined as the minimum concentration required to restore cell viability to near-normal levels- was determined to be 81.5 µg/mL for β-glucan, 83 µg/mL for CM-β-glucan, and 8 µg/mL for piroxicam. Importantly, all EAIC values were significantly lower than their respective IC₅₀ values, indicating that each treatment exhibits anti-inflammatory effects at concentrations well below cytotoxic thresholds, thus confirming their favorable safety profiles.

For β-glucan TI was 2.94% while CM-β-glucan showed a TI of 3.64%. This clearly indicate that β-glucan has a broader safety margin than CM-β-glucan, as its effective concentration is further below its cytotoxic threshold.

To investigate the mechanism behind the anti-inflammatory activity, qPCR was performed to measure the mRNA expression of key pro-inflammatory cytokines- TNF-α and IL-6- in control and LPS-stimulated cells, with and without treatment. LPS stimulation alone significantly increased the expression of all four cytokines: TNF-α (5.1-fold) and IL-6 (18.3-fold), confirming a robust inflammatory response (Fig. 7-B). Piroxicam treatment reduced these levels to 1.53 (70% reduction) and 1.8-fold (90% reduction), respectively.

β-glucan exhibited the strongest inhibitory effect on cytokine expression, decreasing TNF-α levels to 1.3-fold (74.5% reduction) and IL-6 to 3.5-fold (80.8% reduction). This indicates superior performance compared to the reference drug in suppressing TNF-α and a slightly lower effect in reducing IL-6. CM-β-glucan also showed notable anti-inflammatory activity, with expression levels reduced to 2.09-fold (59.01% reduction) for TNF-α and 3.6-fold (80.3% reduction) for IL-6. Although its effect was somewhat lower than that of piroxicam, it still achieved a substantial downregulation of both cytokines, demonstrating meaningful therapeutic potential.

These findings confirm that both β-glucan and CM-β-glucan effectively downregulate pro-inflammatory cytokine expression, highlighting their strong potential as anti-inflammatory agents with favorable safety profiles. Their performance, in terms of cytokine suppression and therapeutic index, was comparable to or even exceeded that of the standard drug piroxicam. This underscores their promise as promising candidates for future pharmaceutical development in the treatment of inflammation-related conditions.

In this study, β-glucan achieved a reduction of 74.5% in TNF-α and 80.8% in IL-6 expression, surpassing the TNF-α suppression reported by Jedinak et al.^53^ for *Pleurotus ostreatus*-derived β-glucan (62%), though showing slightly lower efficacy in IL-6 reduction compared to their reported 93%. These differences may be due to structural or source-related variations, highlighting how β-glucan origin influences its anti-inflammatory efficacy.

### Wound healing

**Fig. 8:**
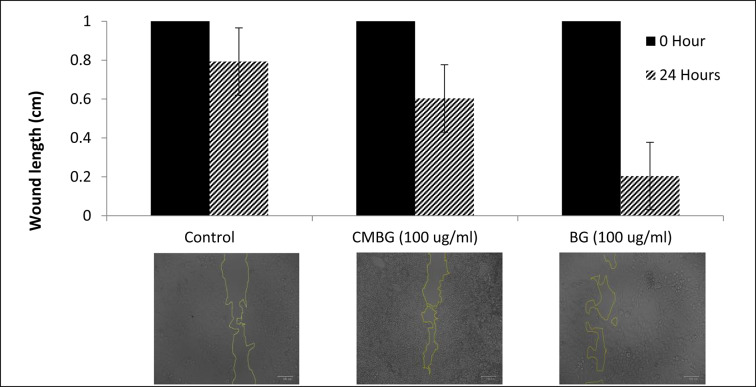
Wound length (cm) of control, CM-β-glucan and β-glucan after 24 h.

Wound closure was evaluated by monitoring the healing of a scratch created in a cell monolayer over a 24-hour period, under both control conditions and treatments with β-glucan and CM-β-glucan. As illustrated in Fig. 8, the initial wound width was identical across all groups at 0 h (10303 μm). After 24 h, the untreated control group exhibited a wound gap of 7926 μm, indicating 23.07% closure (migration distance). In contrast, treatment with β-glucan resulted in a markedly enhanced healing response, reducing the gap to 2041 μm- corresponding to 80.19% closure. CM-β-glucan also facilitated wound closure but to a lesser extent, with a residual gap of 6029.7 μm and 41.47% closure.

The enhanced wound closure observed with β-glucan treatment in this study (80.19% migration distance after 24 h) demonstrates superior efficacy compared to previously reported fungal β-glucans. For instance, Seo et al.^28^ reported only 60% migration following 24-hour treatment with β-glucan derived from *Schizophyllum commune* in a similar scratch wound assay, while β-glucan from black yeast achieved 90% migration. These findings position our β-glucan as a comparably potent agent, highlighting its strong potential in promoting epithelial regeneration. These results clearly indicate that β-glucan significantly accelerates fibroblast migration and proliferation compared to both the control and CM-β-glucan-treated groups. The superior wound healing performance of β-glucan complements its stronger antioxidant capacity, as previously demonstrated in the DPPH radical scavenging assay. Taken together, the enhanced antioxidant and wound healing properties of β-glucan highlight its dual therapeutic potential. Its ability to protect against oxidative stress and promote tissue regeneration positions it as a promising candidate for biomedical applications, particularly in skin repair and wound care formulations.

## Conclusions

This study explores the isolation of yeasts from mangrove aerial roots targeting strains that are β-glucan producers. *Candida tropicalis* AUMC 15,533 was selected for that purpose as it outperformed *Saccharomyces cerevisiae* with a yield of 17.11% of fresh cell weight. NMR and FT-IR analyses confirmed the structural integrity and purity of the extracted β-glucan. Using Plackett–Burman’s statistical experimental design, key factors for β-glucan production were identified as glucose, yeast extract, shaking speed, and incubation time.

Pharmaceutical evaluations show that native β-glucan was more effective than its carboxymethylated derivative (CM-β-glucan) in antioxidant activity and wound healing. Native β-glucan had a superior radical scavenging efficacy (IC₅₀ = 18.31 µg/mL) and achieved 80.19% wound closure in 24 h, compared to 39.29 µg/mL and 41.47% closure for CM-β-glucan and indicated by reducing LPS-induced TNF-α (74.5% and 59.01%) and IL-6 (80.8% and 80.3%) in WISH cells. Both retained favorable safety profiles below cytotoxic levels, but native β-glucan showed a broader safety margin (TI = 2.94%) than CM-β-glucan (TI = 3.64%), indicating its potential as a safer option for biomedical applications.

These findings collectively highlight the potential of *C. tropicalis*- derived β-glucan as a versatile bioactive compound with pharmaceutical applications. Future studies should focus on elucidating the molecular mechanisms underlying its therapeutic effects and exploring its scalability for industrial and clinical applications.

## Supplementary Information

Below is the link to the electronic supplementary material.

Supplementary Material 1

## Data Availability

All data supporting the findings of this study are available within the paper and its Supplementary Information. Any additional data can be obtained from the corresponding author upon request.

## References

[1] Connell, L. et al. Diversity of soil yeasts isolated from South Victoria Land, Antarctica. *Microb. Ecol.* **56**, 448–459. 10.1007/s00248-008-9363-1 (2008). 18253776 10.1007/s00248-008-9363-1

[2] Chi, Z. et al. Production, characterization and gene cloning of the extracellular enzymes from the marine-derived yeasts and their potential applications. *Biotechnol. Adv.* **27**, 236–255. 10.1016/j.biotechadv.2009.01.002 (2009). 19215727 10.1016/j.biotechadv.2009.01.002

[3] Sarkar, S., Pramanik, A., Mitra, A. & Mukherjee, J. Bioprocessing data for the production of marine enzymes. *Mar. Drugs*. **8**, 1323–1372. 10.3390/md8041323 (2010). 20479981 10.3390/md8041323 PMC2866489

[4] Yuan, H., Lan, P., He, Y., Li, C. & Ma, X. Effect of the modifications on the physicochemical and biological properties of β-glucan—a critical review. *Molecules (Basel Switzerland)* 25 (2020). 10.3390/molecules25010057 PMC6983044 31877995

[5] Edo, G. I. et al. Beta-glucan: An overview in biological activities, derivatives, properties, modifications and current advancements in food, health and industrial applications. *Process Biochem.* **147**, 347–370. 10.1016/j.procbio.2024.09.011 (2024).

[6] Ruiz-Herrera, J. *in Structure, Synthesis, and Assembly* **203** (CRC, 2012).

[7] Bowman, S. & Free, S. The structure and synthesis of the fungal cell wall. *BioEssays: News and Rev. Mol. CellularDev. Biol.* **28**, 799–808 10.1002/bies.20441 (2006). 10.1002/bies.20441 16927300

[8] Shahinian, S. & Bussey, H. beta-1,6-Glucan synthesis in Saccharomyces cerevisiae. *Mol. Microbiol.* **35**, 477–489. 10.1046/j.1365-2958.2000.01713.x (2000). 10672173 10.1046/j.1365-2958.2000.01713.x

[9] Ruel, K. & Joseleau, J. P. Involvement of an Extracellular Glucan Sheath during Degradation of Populus Wood by Phanerochaete chrysosporium. *Appl. Environ. Microbiol.* **57**, 374–384 (1991). 16348406 10.1128/aem.57.2.374-384.1991 PMC182720

[10] Ahmad, A., Anjum, F. M., Zahoor, T., Nawaz, H. & Dilshad, S. M. R. Beta Glucan: A Valuable Functional Ingredient in Foods. *Crit. Rev. Food Sci. Nutr.* **52**, 201–212. 10.1080/10408398.2010.499806 (2012). 22214441 10.1080/10408398.2010.499806

[11] Zhu, F., Du, B. & Xu, B. A critical review on production and industrial applications of beta-glucans. *Food Hydrocoll.* **52**, 275–288. 10.1016/j.foodhyd.2015.07.003 (2016).

[12] Douglas, C. M. Fungal beta(1,3)-D-glucan synthesis. *Med. Mycol.* **39** (Suppl 1), 55–66. 10.1080/mmy.39.1.55.66 (2001). 11800269 10.1080/mmy.39.1.55.66

[13] Latgé, J. P. The cell wall: a carbohydrate armour for the fungal cell. *Mol. Microbiol.* **66**, 279–290. 10.1111/j.1365-2958.2007.05872.x (2007). 17854405 10.1111/j.1365-2958.2007.05872.x

[14] Elliott, J. C. *Extracting (1–3/1–6)-β-Glucans from Saccharomyces cerevisiae: A Fungal Immunotherapeutic* (East Tennessee State University, 2016).

[15] Asare, S. O. *Optimized Acid/Base Extraction and Structural Characterization of β-glucan from Saccharomyces Cerevisiae* (East Tennessee State University, 2015).

[16] Schiavone, M. et al. A combined chemical and enzymatic method to determine quantitatively the polysaccharide components in the cell wall of yeasts. *FEMS Yeast Res.* **14**, 933–947 (2014). 10.1111/1567-1364.12182 25041403

[17] Waksman, S. A. A method for counting the number of fungi in the soil. *J. Bacteriol.* **7**, 393–341 (1922). 10.1128/jb.7.3.339-341.1922 PMC378974 16558961

[18] Ding, J., Yufang, W., Shanbai, X., Zhao, S. & Huang, Q. Optimised methodology for carboxymethylation of (1 -> 3)-beta-D-glucan from Yeast (Saccharomyces cerevisiae) and promotion of mechanical activation. *Int. J. Food Sci. Technol.* **48**10.1111/j.1365-2621.2012.03181.x (2013).

[19] Pitt, J. & Hocking, A. D. Fungi and Food Spoilage. *Springer Nat. Switz. AG*, 524 (2009).

[20] White, T. J., Bruns, T., Lee, S. & Taylor, J. Amplification and direct sequencing of fungal ribosomal RNA genes for phylogenetics. *PCR Protocols: Guide Methods Applic.*, 315–322 (1990).

[21] Vardhan, Sasmal, S. & Mohanty, K. Detoxification of areca nut acid hydrolysate and production of xylitol by Candida tropicalis (MTCC 6192). *Prep. Biochem. Biotechnol.* **54**, 1–12. 10.1080/10826068.2023.2207093 (2023). 37149784 10.1080/10826068.2023.2207093

[22] Wahyudi, D. N., Utama, G. L. & Frediansyah, A. Tofu wastewater recovery for β-glucan production by pichia norvegensis and Candida tropicalis. *Curr. Res. Green. Sustain. Chem.* **10**, 100445. 10.1016/j.crgsc.2025.100445 (2025).

[23] Sandula, J., Machová, E. & Hríbalová, V. Mitogenic activity of particulate yeast beta-(1–>3)-D-glucan and its water-soluble derivatives. *Int. J. Biol. Macromol.* **17**, 323–326. 10.1016/0141-8130(96)81839-3 (1995). 8789333 10.1016/0141-8130(96)81839-3

[24] Lowman, D. et al. New Insights into the Structure of (1→3,1→6)-β-D-Glucan Side Chains in the Candida glabrata Cell Wall. *PloS one* **6**, e27614. 10.1371/journal.pone.0027614 (2011). 22096604 10.1371/journal.pone.0027614 PMC3214063

[25] Brand-Williams, W., Cuvelier, M. E. & Berset, C. Use of a free radical method to evaluate antioxidant activity. *LWT*, 25–30 (1995).

[26] Supino, R. MTT assays. *Methods Mol. biology (Clifton N J)*. **43**, 137–149. 10.1385/0-89603-282-5:137 (1995). 10.1385/0-89603-282-5:137 7550641

[27] Sandhiutami, N. M. et al. In vitro assesment of anti-inflammatory activities of coumarin and Indonesian cassia extract in RAW264.7 murine macrophage cell line. *Iran. J. Basic. Med. Sci.* **20**, 99–106. 10.22038/ijbms.2017.8102 (2017). 28133531 10.22038/ijbms.2017.8102 PMC5243982

[28] Seo, G., Hyun, C., Choi, S., Kim, Y. & Cho, M. The wound healing effect of four types of beta-glucan. *Appl. Biol. Chem.* **62**10.1186/s13765-019-0428-2 (2019).

[29] Liang, C. C., Park, A. Y. & Guan, J. L. In vitro scratch assay: a convenient and inexpensive method for analysis of cell migration in vitro. *Nat. Protoc.* **2**, 329–333. 10.1038/nprot.2007.30 (2007). 17406593 10.1038/nprot.2007.30

[30] Ahmed, I. Occurrence and biodiversity of marine yeast in mangrove ecosystem of Shabi Creek, Gwadar- Pakistan. *Pure Appl. Biol.* **8**10.19045/bspab.2019.80008 (2019).

[31] Salem, H. S., Abouzeid, M., Ghazy, M. & Ibrahim, N. A. Diversity of culturable mycoendophytes in Egyptian Red Sea mangrove *Avicennia marina*. *Egypt. J. Bot.* **65**, 219–231 (2025).

[32] Sarkar, N. et al. Beta-glucans in biotechnology: A holistic review with a special focus on yeast. *Bioengineering* **12** (2025). 10.3390/bioengineering12040365 PMC12024604 40281725

[33] Sudiana, G., Kuswendi, H., Yunivita, V. & Roostita, L. B. The potential of Sß-glucan from Saccharomyces cerevisiae cell wall as anti cholesterol. *J. Anim. Health Prod.* **9**10.17582/journal.jahp/2021/9.1.72.77 (2020).

[34] Sukumaran, V., Lowman, D., P, T., Philip, R. & S. & Marine yeast glucans confer better protection than that of baker’s yeast in Penaeus monodon against white spot syndrome virus infection. *Aquac. Res.* **41**, 1799 (2010).

[35] Zaky, A., Tucker, G., Daw, Z. & Du, C. Marine yeast isolation and industrial application. *FEMS Yeast Res.* **14**10.1111/1567-1364.12158 (2014). 10.1111/1567-1364.12158 PMC4262001 24738708

[36] Vidya, P. S. Yeast Diversity in the Mangrove Sediments of North Kerala, India. *Eur. J. Biol.* **81**, 50–57. 10.26650/EurJBiol.2022.1027475 (2022).

[37] Utama, G. L., Suraloka, M. P. A., Rialita, T. & Balia, R. L. Antifungal and aflatoxin-reducing activity of β-glucan Isolated from Pichia norvegensis Grown on Tofu Wastewater. *Foods (Basel Switzerland)* **10**10.3390/foods10112619 (2021). 10.3390/foods10112619 PMC8618602 34828900

[38] Bikmurzin, R., Bandzevičiūtė, R., Maršalka, A., Maneikis, A. & Kalėdienė, L. FT-IR method limitations for β-glucan analysis. *Molecules (Basel Switzerland)*. **27**, 4616 (2022). 35889491 10.3390/molecules27144616 PMC9318380

[39] Baeva, E. et al. Evaluation of the cultivated mushroom Pleurotus ostreatus basidiocarps using vibration spectroscopy and chemometrics. *Appl. Sci.* **10**, 8156 (2020).

[40] Abdel Ghany, K. et al. Description of isolated LAB producing β-glucan from Egyptian sources and evaluation of its therapeutic effect. *Int. J. Pharmacol.* **12**, 801–811. 10.3923/ijp.2016.801.811 (2016).

[41] Li, J. et al. Anti-tumor and anti-metastasis of water-soluble sulfated β-glucan derivatives from Saccharomyces cerevisiae. *Carbohydr. Polym.* **344**, 122466. 10.1016/j.carbpol.2024.122466 (2024). 39218533 10.1016/j.carbpol.2024.122466

[42] Khadam, A. A., Salman, J. A. S. & Hijri, M. Determination the Optimum Conditions for β-glucan Production Extracted from Saccharomyces cerevisiae. *Al-Mustansiriyah J. Sci.* **34**, 32–43. 10.23851/mjs.v34i2.1298 (2023).

[43] Varelas, V., Sotiropoulou, E., Karambini, X., Liouni, M. & Nerantzis, E. T. Impact of Glucose Concentration and NaCl Osmotic Stress on Yeast Cell Wall β-d-Glucan Formation during Anaerobic Fermentation Process. *Fermentation* **3**, 44 (2017).

[44] van der Merwe, R. et al. High salinity tolerance of the Red Sea coral Fungia granulosa under desalination concentrate discharge conditions: an in situ photophysiology experiment. **1**, 10.3389/fmars.2014.00058 (2014).

[45] Sudhir, S. & Arunprasath, A. Sankara Vel, V. A critical review on adaptations, and biological activities of the mangroves. *J. Nat. Pesticide Res.* **1**, 100006. 10.1016/j.napere.2022.100006 (2022).

[46] Gunasekaran, S. & Poorniammal, R. Optimization of fermentation conditions for red pigment production from *Penicillium* sp. under submerged cultivation. *Afr. J. Biotechnol.* **7**, 1894–1898. 10.5897/AJB2008.000-5037 (2008).

[47] Chysirichote, T., Sakoolkaew, P. & Klinthong, W. Correlation of oxygen consumption with growth and red pigment production of Monascus purpureus in solid state fermentation of rice. *Int. Food Res. J.* **31**, 477–485. 10.47836/ifrj.31.2.18 (2024).

[48] Kath, F. & Kulicke, W. M. Mild enzymatic isolation of mannan and glucan from yeast Saccharomyces cerevisiae. **268**, 59–68, (1999).

[49] Zhang, J. et al. Effects of L.plantarum dy-1 fermentation time on the characteristic structure and antioxidant activity of barley β-glucan in vitro. *Curr. Res. food Sci.* **5**, 125–130. 10.1016/j.crfs.2021.12.005 (2022). 35036932 10.1016/j.crfs.2021.12.005 PMC8749382

[50] Fernandes, P. A. R. & Coimbra, M. A. The antioxidant activity of polysaccharides: A structure-function relationship overview. *Carbohydr. Polym.* **314**, 120965. 10.1016/j.carbpol.2023.120965 (2023). 37173007 10.1016/j.carbpol.2023.120965

[51] An, J. et al. Organic Functional Groups and Their Substitution Sites in Natural Flavonoids: A Review on Their Contributions to Antioxidant, Anti-Inflammatory, and Analgesic Capabilities. *Food Sci. Nutr.* **13**, e70191. 10.1002/fsn3.70191 (2025). 40313799 10.1002/fsn3.70191 PMC12041660

[52] Chen, L. et al. Structural characterization and antioxidant activity of β-glucans from highland Barley obtained with ultrasonic-microwave-assisted extraction. *Molecules (Basel Switzerland)*. **29**10.3390/molecules29030684 (2024). 10.3390/molecules29030684 PMC10856557 38338428

[53] Jedinak, A., Dudhgaonkar, S., Wu, Q., Simon, J. & Sliva, D. Anti-inflammatory activity of edible oyster mushroom is mediated through the inhibition of NF-κB and AP-1 signaling. *Nutr. J.* **10**, 52. 10.1186/1475-2891-10-52 (2011). 21575254 10.1186/1475-2891-10-52 PMC3120742

