# Supplementary material for: Wound-healing, anti-inflammatory, and antioxidant activities of β-glucan from red sea-mangroves-associated Candida tropicalis
Source: Sci Rep. 2026 Apr 1;16:11241. doi: 10.1038/s41598-026-42067-0 (PMC13046940; doi:10.1038/s41598-026-42067-0)
Supplement: Supplementary file 1 — Supplementary Material 1 [file 41598_2026_42067_MOESM1_ESM.pdf]

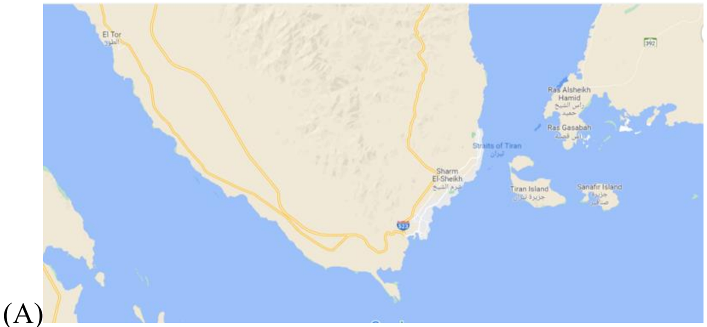

(A)

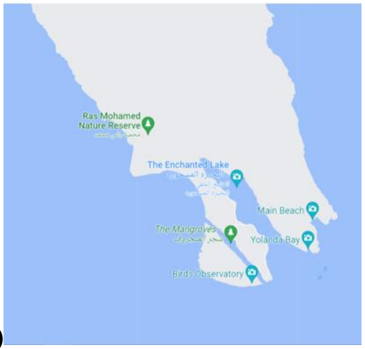

(B)

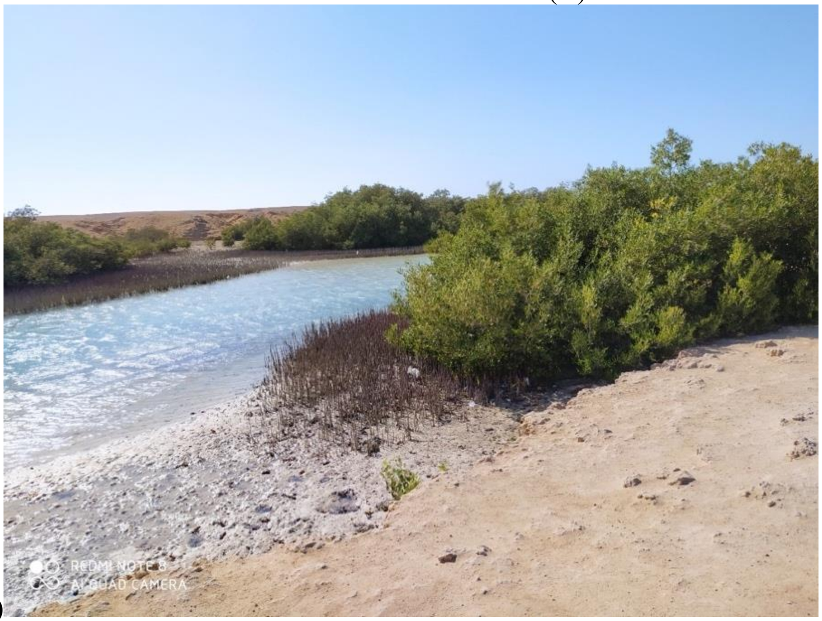

(C)

Supplementary Fig. S1: Site of samples collection A) general map of Ras Mohammed B) A closeup map showing the Hidden Bay C) The Hidden Bay and the Mangrove shrubs.

Supplementary Table S2: High levels and low levels of each factor used in Placket-Burman experimental design.

Factor Number	Ingredients	Low Level (-)	High Level (+)
X1	Glucose (g/L)	10	40
X2	Yeast extract (g/L)	5	20
X3	Peptone (g/L)	10	40
X4	pH	5	7
X5	Shaking (rpm)	0	200
X6	Seawater %	100	0
X7	Incubation period (days)	2	5

Supplementary Table S3: Placket-Burman design for 7 factors.

Trials	X1	X2	X3	X4	X5	X6	X7
1	+	-	-	+	-	+	+
2	+	+	-	-	+	-	+
3	+	+	+	-	-	+	-
4	-	+	+	+	-	-	+
5	+	-	+	+	+	-	-
6	-	+	-	+	+	+	-
7	-	-	+	-	+	+	+
8	-	-	-	-	-	-	-
9	0	0	0	0	0	0	0

Supplementary Table S4: Primers' sequence

Gene	Primer
**TNF-α**	F-5′-CTCTTCTGCCTGCTGCACTTTG-3′
	R, 5′-ATGGGCTACAGGCTTGTCACTC-3′
**IL-6**	F, 5′-TGAACTCCTTCTCCACAAGCG-3′
	R, 5′-TCTGAAGAGGTGAGTGGCTGTC-3′
	R, 5′-TGCTTTGCGTTGGACATTCAAGTC-3′

Supplementary Table S5: Verification experiment for optimization.

Trials	Glucose	Yeast Extract	Peptone	pH	Shaking	Sea Water	Time	Glucan (g/100mL)
**Optimized**	1	1	-1	-1	1	-1	1	0.89
**Anti-optimized**	-1	-1	1	1	-1	1	-1	0.24
**Basal**	0	0	0	0	0	0	0	0.3

Supplementary Table S6: Regression and ANOVA Analysis

Regression Statistics		**ANOVA**				
**Multiple R**	0.999976	***df***	***SS***	***MS***	***F***	***Significance F***
**R Square**	0.999953	7	0.11795	0.01685	3033	0.01398
**Adjusted R Square**	0.999623	1	5.56E-06	5.56E-06		
**Standard Error**	0.002357	8	0.117956			
**Observations**	9					

Supplementary Table S7: Inhibitory concentrations of DPPH (IC50) value by ascorbic acid, β-Glucan and CM-β-Glucan

Samples	DPPH (IC_{50}) μg/mL
**Ascorbic**	3.44±0.01
**β-Glucan**	18.31
**CM-β-Glucan**	39.29

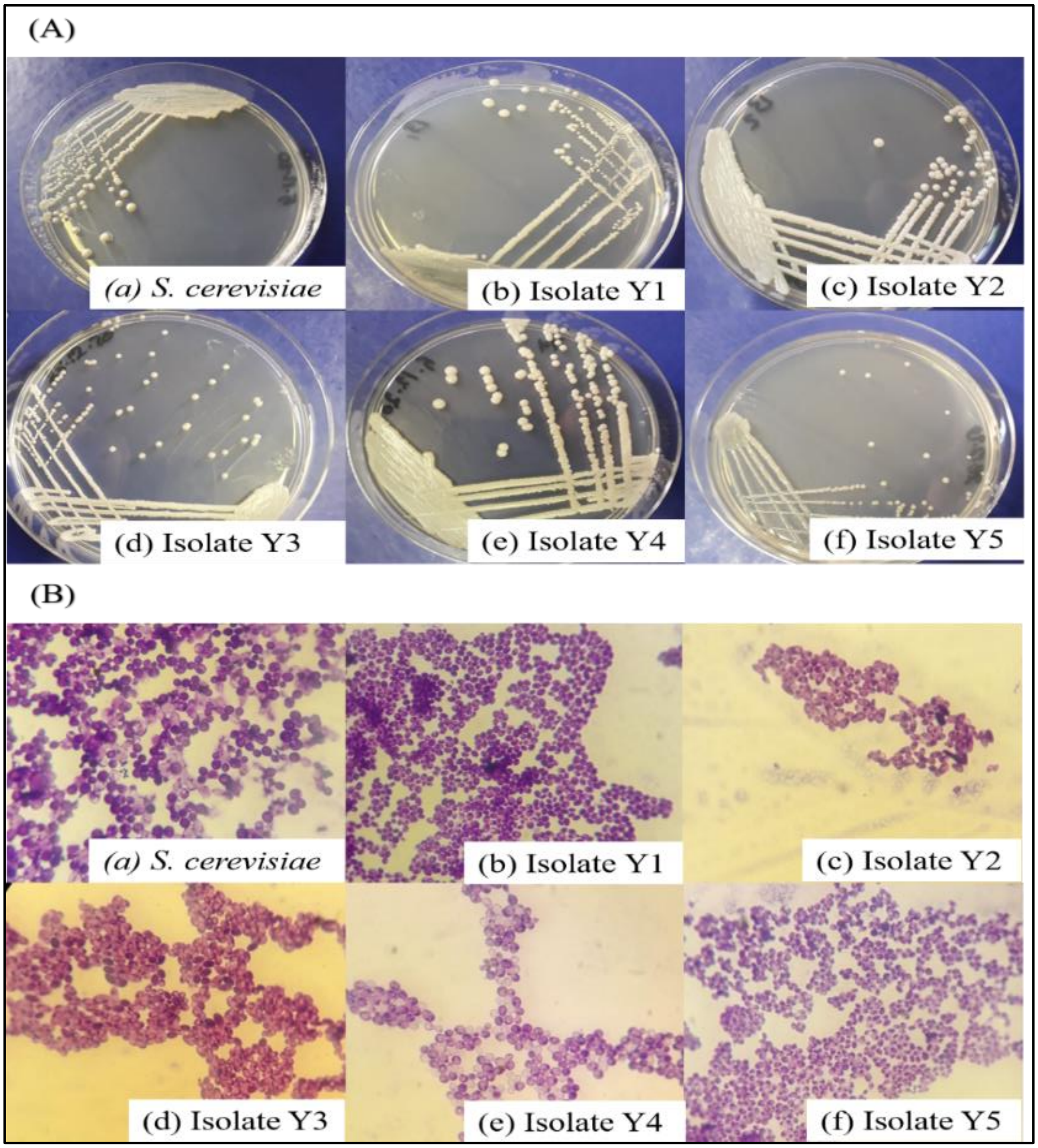

Supplementary Fig. S8: **(A)** Colony morphology on SD agar: (a) *S. cerevisiae*, (b) Y_1, (c) Y_2, (d) Y_3, (e) Y_4, (f) Y_5. **(B)** All six isolates stained with Crystal Violet: (a) *S. cerevisiae*(b) Y_1, (c) Y_2, (d) Y_3, (e) Y_4, (f) Y_5

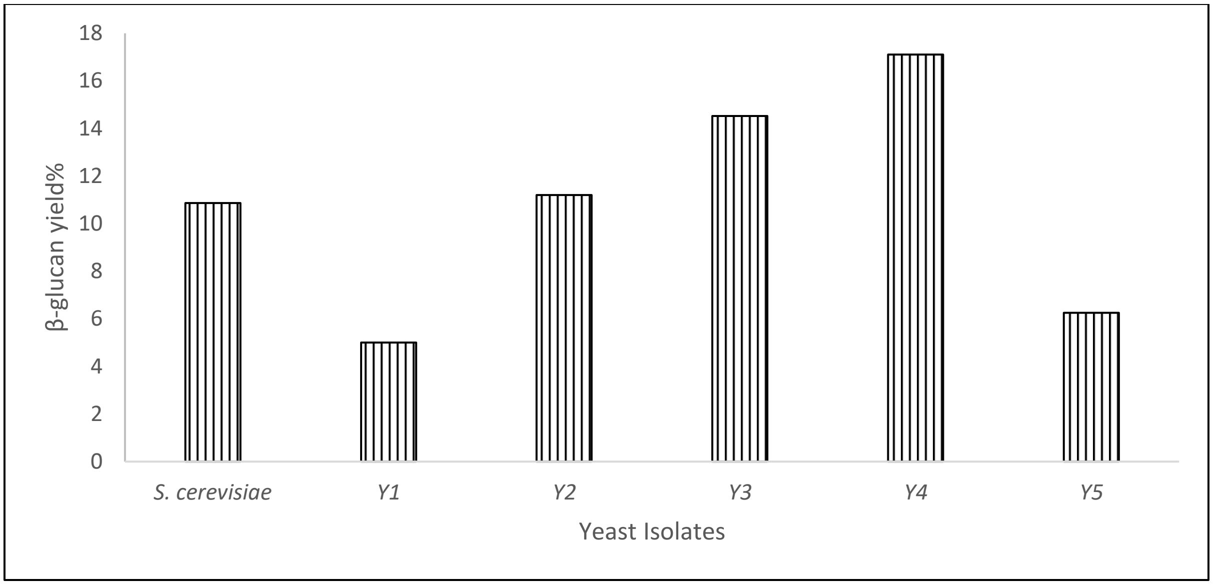

Supplementary Fig. S9: β-glucan yield percentages extracted from each yeast isolates**.**

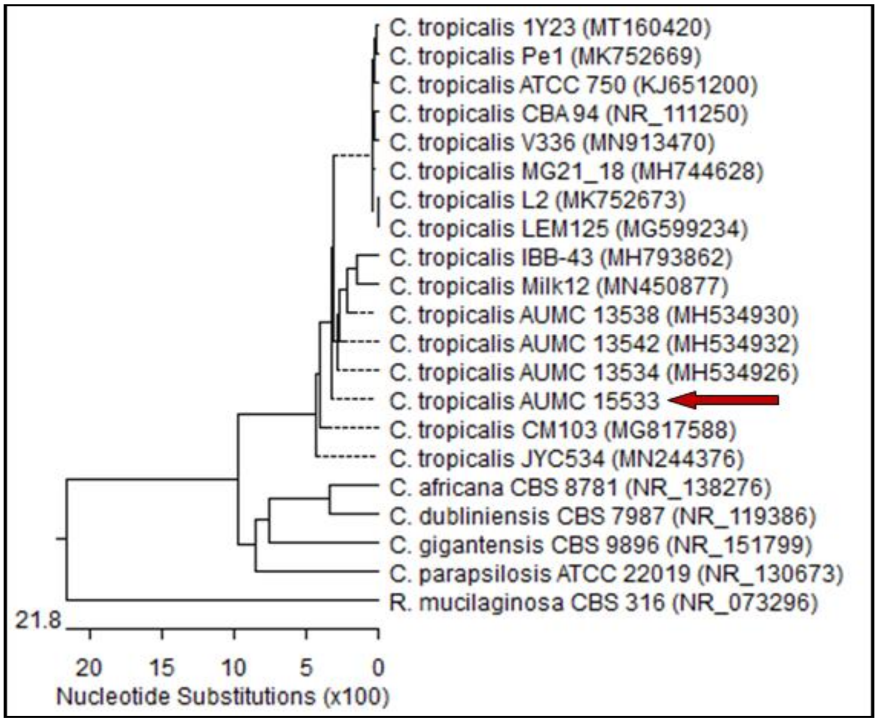

Supplementary Fig. S10: Phylogenetic tree based on ITS sequences of rDNA of isolate Y4 identified as *Candida tropicalis* AUMC15533 (arrowed) aligned with closely related strains. *Rhodotorula mucilaginosa* represents an outgroup strain.
